# Outcomes of Social Egg Freezing: A Cohort Study and a Comprehensive Literature Review

**DOI:** 10.3390/jcm12134182

**Published:** 2023-06-21

**Authors:** Pragati Kakkar, Joanna Geary, Tania Stockburger, Aida Kaffel, Julia Kopeika, Tarek El-Toukhy

**Affiliations:** Assisted Conception Unit, Guy’s and St Thomas’ Hospital NHS Foundation Trust, London SE1 9RT, UK

**Keywords:** social egg freezing, elective oocyte cryopreservation, live birth rate, vitrification, in-vitro fertilization, pregnancy

## Abstract

The purpose of this study is to evaluate the live birth outcome following oocyte thaw in women who underwent social egg freezing at Guy’s Hospital, alongside a detailed published literature review to compare published results with the current study. A retrospective cohort study was conducted between January 2016 and March 2022 for all women who underwent egg freezing during this period. Overall, 167 women had 184 social egg freezing cycles. The mean age at freeze was 37.1 years and an average of 9.5 eggs were frozen per retrieval. In total, 16% of the women returned to use their frozen eggs. The mean egg thaw survival rate post egg thaw was 74%. The mean egg fertilisation rate was 67%. The pregnancy rate achieved per embryo transfer was 48% and the live birth rate per embryo transfer was 35%. We also noted that irrespective of age at freezing, a significantly high live birth rate was achieved when the number of eggs frozen per patient was 15 or more. Despite the rapid increase in social egg freezing cycles, the utilisation rate remains low. Pregnancy and live birth rate post thaw are encouraging if eggs are frozen at a younger age and if 15 eggs or more were frozen per patient.

## 1. Introduction

Oocyte cryopreservation for non-medical reasons, known as social egg freezing, has enhanced the reproductive autonomy of women by allowing the capacity to delay childbearing and preserve the possibility of maintaining a biological relation with future offspring [1].

There has been a considerable increase in the number of oocyte cryopreservation cycles in last decade. In the UK, a 460% increase in oocyte cryopreservation cycles has been reported between 2010 and 2016 [2]. Similarly, an 880% and a 507% increase between 2010 and 2015 have been reported in the USA and Australia and New Zealand, respectively [3,4]. Advancements in cryopreservation techniques, particularly oocyte vitrification (or ultra-rapid freezing) in last decade, have led to remarkable improvements in the efficacy of oocyte cryopreservation, post-thaw oocyte survival, fertilisation, and pregnancy rates, and therefore broad acceptability and applicability of this reproductive option [1,5]. Additionally, the Human Fertilization and Embryology Authority (HFEA) in the U.K. approved the use of frozen oocytes in 2000, highlighting that using frozen eggs in infertility treatment is sufficiently safe [6] and in 2013, the American Society of Reproductive Medicine (ASRM) Ethics Committee removed the experimental label from planned oocyte cryopreservation [2,6,7].

Since then, a vast literature has been published covering various aspects of oocyte cryopreservation. As undergoing social oocyte freezing is a sensitive experience bearing a complex psychological and social impact on patients, various studies have evaluated the aspect of women’s experiences with this process including making decisions and regret scales, and have concluded that there is a need for adequate counselling regarding benefits, risks, alternative reproductive options, and emotional support throughout the oocyte freezing process [8,9,10]. One of the latest studies has also explored the perspectives of patients and providers about social egg freezing, highlighting the decision to undergo this process is complex and individual patient values play a significant role. They concluded there is disconnect between providers and patients in their views on how to navigate decision making, and these should be addressed to improve shared decision making [11]. To help women with this decision process, a decision aid tool has been developed that aims to investigate the effects of an elective egg freezing decision aid on decision-related outcomes (e.g., decisional conflict, informed choice, and regret), expecting better and satisfactory decisions by women [4]. Studies also evaluated reproductive intentions and actions after oocyte cryopreservation [12].

As a result, there is growing need to have contemporary reliable data to raise awareness amongst women about the expected outcomes post thaw, following social egg freezing [13].

To date, studies reporting outcomes in women returning to use their cryopreserved oocytes after social egg freezing are scarce [14,15,16]. Hence, we conducted this study with an objective to evaluate the outcomes of egg thaw at Guy’s Hospital, following social egg freezing and to compare our results with a comprehensive literature review of published data in order to provide evidence-based contemporaneous information to help women make an informed decision when planning to embark on social egg freezing.

## 2. Materials and Methods

### 2.1. Local Data Collection

A data search of electronic medical records was conducted to identify all women who underwent social egg freezing between January 2016 and March 2022, at Guy’s Hospital Assisted Conception Unit, an inner city London-based IVF Centre.

The search included all women who underwent social egg freezing using vitrification technology during this period. The exclusion criteria included women undergoing fertility preservation for medical or oncological reasons and those who had egg freezing due to their partner not being able to produce sperm on the day of oocyte retrieval. Women who returned to start on oocyte thaw cycle were identified up to March 2023. The variables collected included demographic data at the time of egg freezing, number of oocytes frozen, duration from oocyte freeze to thaw, post-thaw oocyte survival and fertilisation rates, numbers of embryos transferred and frozen, and pregnancy and live birth rate. All the patient data were analysed by two independent reviewers (PK and TS).

### 2.2. Data and Statistical Analysis

The variables collected were transferred to an Excel sheet for analysis. The age at oocyte freeze, number of oocytes frozen, duration from oocyte freeze to thaw, post-thaw oocyte survival and fertilisation rates, and numbers of embryos transferred and frozen were categorised and assessed by age-wise distribution of less than 35 years, the age group between 35 years and 37 years, and 38 years and above. Pregnancy and live birth rates were stratified by age younger than 38 and age 38 years and above at the time of cryopreservation and reported using descriptive statistics.

Clinical pregnancy rate was defined as the presence of a foetal cardiac pulsation on ultrasound scanning 3 or more weeks after a positive pregnancy test. Live birth rate was defined as a delivery of a live born baby at 24 weeks or beyond.

Categorical variables were analysed using the Fischer’s exact or chi-squared test where appropriate.

### 2.3. Treatment Protocol

Treatment for all the patients were analysed in detail. All patients used short antagonist protocol with agonist trigger as described previously by a few studies [17,18]. This regimen was especially beneficial for women with a high ovarian reserve, suggesting that antagonist IVF therapy can allow for a safer and more effective clinical outcome. An individualistic approach was adopted while prescribing medications for each patient on the basis of their antral follicle count, age, and anti-mullerian hormone level. For all protocols, gonadotropins (recombinant follicle-stimulating hormone (Gonal-F), human menopausal gonadotropins (Menopur), or Gonal-F and Menopur) were administered on day 2 or 3 of menstrual cycle following a baseline ultrasound scan, and follicular growth was monitored by a transvaginal ultrasound scan and serum estradiol levels, where necessary. On day 6 of stimulation, the gonadotropin-releasing hormone antagonist (Cetrotide or Fyramedal) was added to avoid premature luteinization, follicle maturation arrest, and asynchrony of oocyte maturation. When 3 follicles reached a measurement of 18 mm or above, the gonadotropin-releasing hormone agonist (Buserelin 0.5 mL) was used for the trigger of final follicular maturation with a planned oocyte aspiration scheduled for 36 h after administration. Oocyte retrieval was performed via ultrasound-guided transvaginal aspiration under sedation. Further assessment of oocyte suitability for freezing was undertaken by followed by analysis of oocytes by embryologist and only Methaphase II oocytes were deemed suitable for vitrification. All oocytes were cryopreserved using the vitrification method.

Once oocytes were obtained and denuded within an hour of egg coollection, they were placed in a high-protein buffer medium for 1 min. Oocytes were gradually exposed to increasing concentration of equilibration solution over 4 min. Following that, they were moved into the third equilibration solution for 6–10 min. After that, oocytes were transferred to the vitrification solution for a maximum of 1 min until they were loaded on to a cryolock device and plunged into liquid nitrogen [19]. Embryologists trained in oocyte cryopreservation and thawing carried out all laboratory procedures. When patients returned to use their cryopreserved oocytes, intracytoplasmic sperm injection was used to fertilize thawed oocytes. Embryos were cultured to day 3 or 5 of development for transfer depending upon their grading (Gardner and Schoolcraft, [20]). The frozen embryo transfers were then carried out after preparing the endometrial lining with medications or in a natural cycle. The medicated frozen embryo transfer cycle comprised giving oral oestrogen or transdermal oestrogen patches or a combination of both for 14 days, starting from day 2 of the period cycle, followed by a departmental scan to check the thickness of the endometrial lining. Once the lining was 7 mm or more, progesterone pessaries were started for 3 or 5 days, depending on the grade of embryo. The embryo transfer was then performed under ultrasound guidance. Patients were asked to conduct the pregnancy test and were advised to contact the unit to report the outcome. If they were pregnant, a departmental scan was arranged for them after 7 weeks of gestation to confirm the viability. They were discharged from the unit to carry on their further care with obstetric department. If the pregnancy test was negative, they were advised to stop their medications and attend a follow up consultation with consultant to discuss the cycle and further steps of treatment.

### 2.4. Literature Review

A literature search was performed on Pubmed and Embase databases using combination terms, keywords “social egg freeze”, “elective egg freeze”, and “oocyte cryopreservation”. The search included all studies published in English language from database inception until December 2022. All relevant published studies were evaluated. For data extraction, the following information was selected: author(s), year of publication, country in which the study was conducted, aim of the study, study design, and main results including live birth rate. After collecting the information, citations were assessed for the suitability of selection by two individuals (PK and TS) and included if they reported a live birth rate following treatment using frozen–thawed eggs retrieved for social reasons.

### 2.5. Study Selection Process

**Figure 1:**
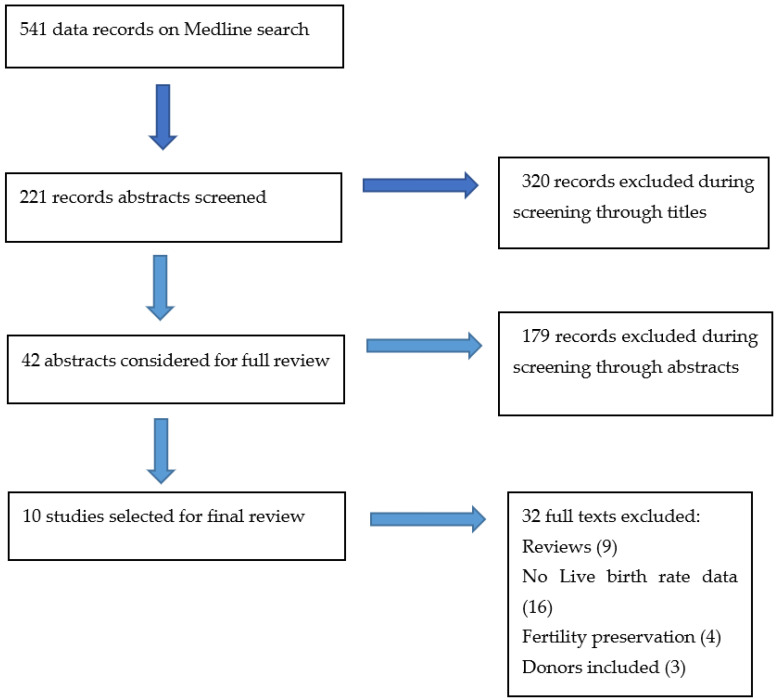
Selection process for the study.

The initial search included 541 articles that were filtered into 221 after review of titles. The abstracts of 221 citations were examined by two independent reviewers (PK and TS) and the full text of 42 articles were selected and assessed for suitability. The most important parameter used to assess the suitability of the articles was live birth rate. Finally, 10 relevant manuscripts were selected for inclusion in our study. Any disagreements between the two reviewers were arbitrated by third reviewer (TET) until a consensus was reached (Figure 1).

## 3. Results

**Figure 2:**
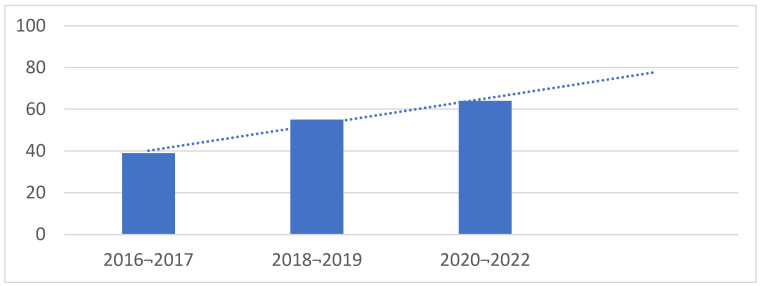
Uniform rise in egg freezing cycles depicted during January 2016–March 2022.

During the period of January 2016–March 2022, 167 women had 184 social egg freezing cycles at Guy’s Hospital Assisted Conception Unit. The mean age at egg freezing in this study was 37.1 years. Figure 2 shows the constant increase in number of social egg freezing cycles during the study period.

**Figure 3:**
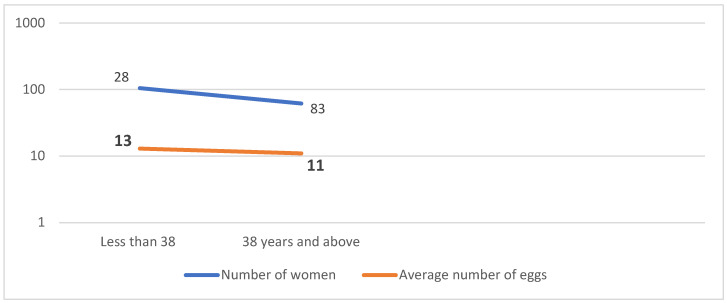
Relationship between number and age of women freezing eggs with average number of eggs frozen.

The mean number of eggs frozen in our study was 9.5 eggs per retrieval, with the highest mean number of eggs frozen in women under 35 years of age (Figure 3). The stimulation protocol used for all the women was short antagonist [14]. 

### 3.1. Outcomes of Thaw Cycles

Overall, 27 (16%) women returned to use their frozen eggs between January 2016 and March 2023. Of those women, 12% were under 35 years of age at the time of egg freeze, 44% were between the age group of 35 to 38, and 44% women were older than 38 years (Table 1).

The average duration of oocyte freezing for women who requested using their eggs was 3.9 years. This duration of storage decreased as the female age at freezing increased, being 5.3 years in women less than 35 years, 3.8 years for women aged between 35 and 38 years of age, and 2.6 years for women over 38 years of age (Table 1).

Among the 27 women who came back to use their eggs, 25 (93%) thawed all their stored eggs, whereas 2 (7%) requested to thaw 50% of their eggs. In total 346, frozen oocytes were thawed. Mean post-thaw survival rate was 74% (257/346). The oocyte survival rate was 68% (119/176) in women younger than 38 years and 81% (138/170) in women 38 years of age and more, indicating age at freezing has no impact on the survival rate of thawed eggs (*p*-value—0.73).

All 257 eggs that survived the thaw were utilised for ICSI. The average fertilisation rate was 172/257 (67%) and the age-wise distribution showed a fertilisation rate of 116/155 (75%) less than age of 38 years, 60/102 (59%) for women with age more than 38 years (Table 1).

The embryo transfer were carried out in 20 cycles and in 7 cycles, no embryos were available for transfer, 3 due to failed fertilisation and 4 due to failure to cleave of fertilised eggs. The total number of transfers that happened among these 20 women was 23. The number of pregnancies achieved per embryo transfer was 11/23 (48%), and the live birth rate per embryo transfer was 35% (8/23). The age-wise distribution showed a pregnancy rate of 56% (9/16) in women less than 38 years of age, and 29% (2/7) in women 38 years and over. The age-wise distribution of live birth rate was 38% (6/16) in women less than 38 years of age, and 29% (2/7) in women in the age group of 38 years and above.

We also noted that if more than 15 eggs were thawed, irrespective of age group, the pregnancy rate was 7/11 (64%) per patient and live birth rate was 5/11 (45%) per patient, compared to less than 15 eggs where pregnancy rate was 4/15 (27%) and live birth rate was 2/15 (13%).

### 3.2. Results from Literature Review

Overall, 10 studies were selected to compare the published literature with our study.

The average age at freezing was quoted in all of them and was calculated to be 37 years from collated data.

All the studies discussed include women requesting usage of their socially frozen eggs. The collated data revealed 1006 women returned to use their eggs out of 8208, giving an average usage of 12% (Table 2).

Out of 10 studies, the oocyte survival rate post thaw was quoted in 4 studies, compared with [15,22,24,27], wherein the number of eggs surviving the thaw were 82% (6462/7904).

Similarly, the oocyte fertilisation rate was comparable in 2 out of 10 studies [15,22] and the collated data from the 2 studies showed an average egg fertilisation rate of 67%. In total, 2 out of 10 quoted fertilisation rate in percentage without exact numbers, however the average percentage of fertilisation rate was similar (67%).

Live birth rate results could be obtained to compare from 7 out of 10 studies, as the numbers could be extracted from them and correlated with our data. We noted that out of 558 women, live births were achieved by 196 women giving an average of 35%. The age-wise distribution was comparable mainly in three studies [15,16,22]; the reason being varied age-group distribution. In total, 24/50 (48%) women achieved live birth and on-going pregnancy in the age group less than 38 years and 20/94 (21%) in the age-group 38 years or older. In three studies stating live birth rate, the data were given as live birth rate per woman. As a result, the comparison with these studies could not be made in terms of live birth rate.

## 4. Discussion

To the best of our knowledge, this study is the first to review the worldwide literature of outcomes of egg thaw cycles following social egg freezing. We compared our post thaw data against the collated worldwide data and combined ours to the existing collated literature, creating a counselling tool for patients embarking on social egg freezing.

It has been indisputably evident from the current data that the demand for social egg freezing has been growing exponentially over the last decade. We anticipate that this rise will continue to occur in future and, hence, more studies are needed to provide adequate information to women considering social egg freezing. 

Various studies conducted worldwide to date have quoted mean ages for egg freeze, giving an overall average age of 37 years when combined, which was similar to our study, highlighting the importance of raising awareness among women about age-related fertility decline and the importance of freezing eggs at an earlier age, to increase the oocyte yield [28,29]. Both ESHRE and Nordic Fertility Society have recommended that the most cost-effective time to undergo egg freezing is prior to 35 years of age when the chance of a live birth following oocyte thawing could reach up to 75% [1,30]. Hence, it is of paramount importance to provide women with realistic individualised advice about their own chances of successful egg freezing raising false hopes [13]. In our study, the live birth rate per cycle of egg thawing was over twice as high in women younger than 38 years compared to women 38 years and older. 

It is also important to note that the 10-year storage limit on frozen eggs in the UK, which may have acted as a disincentive for British women to freeze their eggs early, has now been changed to 55 years, giving women reassurance to freeze their eggs when they are in their most fertile period and take away the fear of having to discard their eggs before they are ready to use them.

The upper age limit noted in our study was 42 years and a similar age limit has been reported in the published literature. Furthermore, it is clear from the existing literature that oocyte freezing after the age of 42 years is unlikely to result in a live birth due to suboptimal ovarian response and higher cycle cancellation rates [1,2,31]. 

Currently, all published data for usage rate show that the average return rate worldwide is low, around 12% (Table 2). Various reasons for this could be women not wanting to start a family without the right partner, preferring to conceive naturally and keeping the frozen eggs as a backup, or not wanting to use a sperm donor. The Human Fertilisation and Embryology Authority (HFEA) data stated that partner status was a major reason for the same [1,32,33]. 

The comparison between our data and existing worldwide studies is outlined in Table 3. These results reveal that even though more women are freezing eggs, very few are actually returning to use them. It is possible that with longer follow up periods, the usage rate could increase and therefore further studies with longer follow up periods are required.

One of the interesting findings of note in our study was that women who were older at the time of egg freeze returned sooner to use their eggs compared to those who froze their eggs at a younger age (Table 1). Similar findings were also reported by Blakemore et al. in 2021 [22]. They stated that patients who were older at the time of cryopreservation thawed their oocytes sooner than those who were younger at the time of cryopreservation (*p* < 0.002), giving an intuitive argument that younger patients at the time of freeze are less likely to use or need to use their oocytes. Some of the reasons for the above could be attributed to the fact that women less than 35 years old may conceive spontaneously [14] or women have to solve different situations in their lives, which initially made them choose elective oocyte cryopreservation [24], etc.

As a result of low usage rates, there has been considerable debate about the cost effectiveness of social egg freezing and about funding for the same [34]. Studies have shown that planned oocyte cryopreservation is cost-effective only if 49–61% of patients return to use their oocytes [35,36], which is a very high return rate compared to the current published literature, 12%. Social egg freezing is privately funded in most countries, including the U.K. The average price for a complete oocyte freezing and thawing cycle, including annual storage fees, could reach over GBP 7000 in UK, between USD 15,000 to 20,000 per cycle (Fertility IQ, 2018) in United States and approximately AUD 8000 per cycle in Australia [1]. Given how expensive the treatment is, along with low usage rates, we strongly recommend that during the first consultation, women should be provided with the details of costs per cycle and the detailed information about usage rates (current versus expected) so that they are able to make an informed decision about their treatment.

One of the other important aspects of the discussion is oocyte survival rate per oocyte thaw, which could be extracted from four studies and was calculated to be around 80% (Table 3). This shows that oocyte survival rate is on average similar worldwide and is not affected by women’s age at egg freezing.

Next, of the total filtered results, the average fertilisation rate in our study and in the existing literature was noted to be 67%; when the data was collated to our study, the results showed the fertilisation rate to be 68% (Table 3), indicating that our results can be generalised reliably to counsel women for social egg freezing.

Quite often, women seeking fertility preservation through social egg freezing are keen to know the likelihood of success based on the number of oocytes obtained and cryopreserved. In our study, it was noted that irrespective of age at freezing, a significantly higher live birth rate was achieved when the number of eggs frozen per patient was 15 or more (*p*-value 0.013), indicating freezing higher numbers of eggs increases the chance of a live birth. Similarly, Cobo et al. in 2018 demonstrated the high efficacy of social egg freezing, as the probability of a live birth increases with the increase in the number of cryopreserved oocytes, depending on the age of the oocyte.

According to the study, patients less than 35 years old with 8–10 oocytes yield a cumulative probability of having a baby of ~30% and 45%, respectively; furthermore, with five additional oocytes, i.e., 15 oocytes success rates rose to ~70%. With ~25 oocytes, the cumulative probability rose to ~95%. Although this is also true for older patients, those aged more than 35 years need more oocytes to match these results [24]. Some studies also concluded approximately 20 oocytes are needed to have around 75% likelihood of achieving at least one child provided that the woman is younger than 38 years [31,37].

Mathematical models have been published that can be used to predict the live birth-rate probability based on the number of cryopreserved oocytes and the woman’s age at the moment of cryostorage. For example, to obtain a 75% CLBR, women at 34, 37, or 42 years need to cryopreserve 10, 20, and 61 eggs, respectively [38].

As the number of oocytes needed to freeze varies widely between studies, this target number should be personalised and individualised in different fertility centres and settings based on each woman’s own ovarian reserve as indicated by her anti-Müllerian hormone (AMH) level and antral follicle count (AFC) measurements on an ultrasound scan as well as each IVF centre’s own experience and success rate data [1].

Furthermore, the average live birth rate following oocyte cryopreservation, in the existing literature, is quoted as 35% per embryo transfer which is comparable to the data from our study at Guy’s Assisted Conception Unit (Table 3). The age-wise distribution shows the live birth rate following egg thaw cycles in our unit as comparable to fresh IVF cycle as per the Human Fertility and Embryology Authority (HFEA) [39]. In 2013, the Practice Committees of the American Society for Reproductive Medicine and the Society for Assisted Reproductive Technology conducted a systematic literature review in order to compare the efficacy (clinical pregnancy and live birth rates) of embryo transfers using fresh or cryopreserved/thawed oocytes. The review states that there is good evidence to suggest that fertilization and pregnancy rates are similar to IVF/ICSI with fresh oocytes when vitrified/warmed oocytes are used as part of IVF/ICSI in young infertility patients and oocyte donors. No increases in chromosomal abnormalities, birth defects, or developmental deficits have been noted in the children born from cryopreserved oocytes. There is also evidence to suggest that livebirth rate and perinatal outcomes are similar in patients after oocyte and embryo cryopreservation [40].

This result is very important in counselling women considering social egg freezing in order to provide some reassurance to them regarding treatment effectiveness and encouraging future outcomes.

Despite of our best efforts, our study does have the limitation of a small sample size, which is similar to the existing published literature. We aim to follow up on future cases in our unit to overcome this.

Our study findings have several important implications for potential patients wanting to freeze their eggs. We believe that these outcomes will be vital to both physicians and patients to help guide them with individualized counselling and provide realistic expectations and adequate information for patients embarking on social egg freezing.

## Figures and Tables

**Table 1:** Summary of the results of the study with their significance.

Variables	Age, Less than 38 Years	Age, 38 Years or Above	*p*-Value
Number of patients returned back (*n* = 27)	15 (56%)	12 (44%)	NA
Mean eggs frozen	12	9	0.41
Average duration of storage (*n* = 27)	5.46 years	3.1 years	0.70
Survival rate	119/176 (68%)	138/170 (81%)	0.73
Fertilisation rate per oocyte injected	116/155 (75%)	60/102 (59%)	0.26
Pregnancy rate per ET	9/16 (56%)	2/7 (29%)	0.22
Live birth rate per ET	6/16 (38%)	2/7 (29%)	0.77

**Table 2:** Summary of the details of the published literature.

Articles	Mean Age	Women Returned	Oocyte Survival	Fertilisation Rate per Oocyte Injected	Fertilisation Rate per Oocyte Injected	Live Birth Rate per ET/per Women
Kasaven et al., 2022 [14]	38	36/373	-	60%	-	30% per ET
Harjee et al., 2022 [21]	36.5	50/556	-	60%	-	65% per ET
Avi Tsafrir et al., 2022 [15]	37.9	57/446	-	-	-	27% per woman
Lh-Jane Yang et al., 2022 [16]	38.1	68/921	-	74%	-	39% per ET
Blakemore et al., 2021 [22]	38.2	88/231	932/1256	70%	642/932	27% per ET
Gurtin et al., 2019 [23]	37.7	46	-	-	-	17% per woman
Wafi et al., 2019 [24]	36	28/138	-	-	-	21% per woman
Cobo et al., 2018 [25]	37.2	641/5289	4891/5830	-	-	39% per ET
Wennberg et al., 2018 [26]	37	38/254	307/393	62%	190/307	37% per ET
Nagy et al., 2017 [27]	36.9	50	332/425	-	-	20% per ET
Average	37.3	1006/8208 (12%)	6462/7904 (82%)	67%	832/1239 (67%)	

**Table 3:** Summary of the outcomes post oocyte thaw and comparison with the published literature.

Outcomes	Current Study	Worldwide Data	Collated Data
Mean age at freezing	37.1 years	37.3 years	37.2 years
Usage rate	16%	1006/8208 (12%)	1031/8375 (12%)
Thaw/survival rate per oocyte frozen	74%	6462/7904 (82%)	6508/8230 (79%)
Fertilisation rate per injected oocyte	67%	832/1239 (67%)	977/1439 (68%)
Live birth rate per ET	35%	196/558 (35%)	203/580 (35%)
Less than 38 years	38%	24/50 (48%)	29/65 (45%)
38 years of age and above	29%	20/94 (21%)	22/101 (22%)

## Data Availability

Data presented in this study are not publically available due to ethical restrictions.
